# A Computational Understanding of Inter-Individual Variability in CYP2D6 Activity to Investigate the Impact of Missense Mutations on Ochratoxin A Metabolism

**DOI:** 10.3390/toxins14030207

**Published:** 2022-03-14

**Authors:** Jean Lou C. M. Dorne, Martina Cirlini, Jochem Louisse, Lorenzo Pedroni, Gianni Galaverna, Luca Dellafiora

**Affiliations:** 1 Scientific Committee and Emerging Risks Unit, European Food Safety Authority, Via Carlo Magno 1A, 43124 Parma, Italy; jean-lou.dorne@efsa.europa.eu; 2 Department of Food and Drug, University of Parma, 43124 Parma, Italy; lorenzo.pedroni@unipr.it (L.P.); gianni.galaverna@unipr.it (G.G.); 3 Wageningen Food Safety Research, P.O. Box 230, 6700 AE Wageningen, The Netherlands; jochem.louisse@wur.nl

**Keywords:** ochratoxin A, CYP2D6, polymorphism metabolism, toxicokinetics, food safety, hazard assessment

## Abstract

Cytochrome P-450 (CYP) enzymes have a key role in the metabolism of xenobiotics of food origin, and their highly polymorphic nature concurs with the diverse inter-individual variability in the toxicokinetics (TK) and toxicodynamics (TD) of food chemicals. Ochratoxin A is a well-known mycotoxin which contaminates a large variety of food and is associated with food safety concerns. It is a minor substrate of CYP2D6, although the effects of CYP2D6 polymorphisms on its metabolism may be overlooked. Insights on this aspect would provide a useful mechanistic basis for a more science-based hazard assessment, particularly to integrate inter-individual differences in CYP2D6 metabolism. This work presents a molecular modelling approach for the analysis of mechanistic features with regard to the metabolic capacity of CYP2D6 variants to oxidise a number of substrates. The outcomes highlighted that a low-frequency CYP2D6 variant (CYP2D6*110) is likely to enhance ochratoxin A oxidation with possible consequences on TK and TD. It is therefore recommended to further analyse such TK and TD consequences. Generally speaking, we propose the identification of mechanistic features and parameters that could provide a semi-quantitative means to discriminate ligands based on the likelihood to undergo transformation by CYP2D6 variants. This would support the development of a fit-for-purpose pipeline which can be extended to a tool allowing for the bulk analysis of a large number of compounds. Such a tool would ultimately include inter-phenotypic differences of polymorphic xenobiotic-metabolising enzymes in the hazard assessment and risk characterisation of food chemicals.

## 1. Introduction

Xenobiotic metabolism involves complex processes and a huge number of xenobiotic-metabolising enzymes such as cytochromes P-450 (CYPs) as phase I enzymes and UDP-glucuronosyltransferases as phase II enzymes. Such xenobiotic-metabolising enzymes have prominent roles in the metabolic fate of a wealth of endogenous and exogenous low-molecular-weight compounds [1,2]. Indeed, CYPs are major ubiquitous phase I enzymes, occurring in thousands of isoforms across bacteria, plants, fungi and animals, and play a key role in the pharmacology and toxicology of a plethora of compounds including pharmaceuticals, pesticides, food and feed additives, food components and contaminants to cite but a few. CYP isoforms are often polymorphic by nature, and such polymorphisms have an impact on their expression and activity profiles and consequently on inter-individual differences in toxicokinetic (TK) and toxicodynamic (TD) processes [3]. In this context, the human CYP2D6 is a member of the CYP2D gene family and a highly polymorphic isoform with more than 146 alleles (referred to as alleles stars, *) described so far (https://www.pharmvar.org, accessed on 4 October 2021) [4,5]. Missense mutations due to single-nucleotide polymorphisms (SNPs) or base deletions or insertions have been associated either to the loss of or reduction in function, or to a functional conservation over certain substrates (as per the PharmVar database; https://www.pharmvar.org, accessed on 4 October 2021). The combinations of allelic variants known as haplotypes have differential arrangements within the human genome with four key phenotypes, namely ultra-rapid, extensive, intermediate and poor metabolisers (UM, EM, IM and PM) [6,7]. Frequencies for CYP2D6 allelic variants are variable across human populations of different geographical origins and even differ within the same population. Such variability in CYP2D6 may have consequences on the TK and TD of a number of xenobiotics [8,9]. The current characterisation of the CYP2D6 metaboliser status has been based on analyses of the activity of allelic variants forming the known haplotypes and provides a useful tool to pinpoint the function of individual genotypes [6]. In this respect, the activity of high-frequency allelic variants has been relatively well characterised, while most low-frequency variants still have unknown or uncharacterised activity profiles (as per the PharmVar database; https://www.pharmvar.org, accessed on 4 October 2021). This data gap represents a hindrance in the prediction of the metaboliser status of subpopulations or individuals harbouring haplotypes with low frequencies and uncharacterised allelic variants. In addition, such data gaps do not allow the integration of CYP2D6 inter-individual variability across world populations in the hazard and risk characterisation of chemicals.

**Figure 1:**
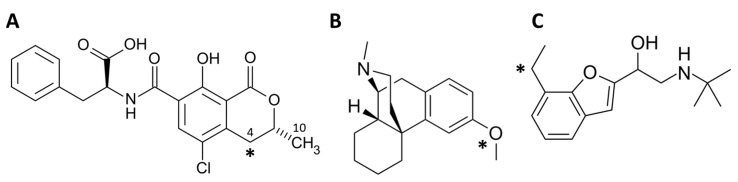
Chemical structure of molecules under analysis (drawn with ChemDraw version 20.1.1; PerkinElmer Informatics). The asterisk indicates the atom undergoing the reaction considered in this study. (**A**) OTA. (**B**) Dextromethorphan. (**C**) Bufuralol.

In this context, the present work provides a computational workflow to model the impact of single-nucleotide polymorphism (SNP) missense mutations, present in a number of low-frequency CYP2D6 variants, on the capacity to oxidise specific substrates while providing comparative results for the reference isoform CYP2D6*1. In this respect, molecular modelling approaches have already proven to successfully model CYP activities, including CYP2D6 and other isoforms. In addition, these approaches provide a useful analytical means to correlate primary CYP protein sequences and their metabolic activities [10,11,12]. Here, this manuscript focuses on the metabolic transformation (oxidation) of ochratoxin A (OTA; Figure 1) by two low-frequency and poorly characterised CYP2D6 allelic variants, namely CYP2D6*122 and CYP2D6*110, compared to the wild-type CYP2D6*1.

In the food safety area, OTA is a well-known mycotoxin commonly present in cereals, coffee, wine, dried fruits, nuts and meat products [13,14] and its relevance to food safety has been unambiguously documented [15]. In addition, OTA’s toxicity has been shown to partly involve bioactivation through hydroxylation, although the major elimination pattern for OTA has been shown to be active transport and renal excretion [16,17]. Nevertheless, several human OTA metabolites have been described, including 4-hydroxy-OTA (4-OH-OTA), 10-hydroxy-OTA (10-OH-OTA), ochratoxin alpha (OTalpha), as well as several conjugate derivatives with glutathione, sulphate or glucuronic acid groups [15,18]. OTalpha is described as the main metabolite resulting from detoxification, and it has been shown to be mostly produced by gut microbiota. However, non-microbial carboxypeptidases have also been described to hydrolyse OTA to OTalpha, including the bovine carboxypeptidase A and the porcine ortholog carboxypeptidase B [15,19,20]. Hydroxylation on the isocoumarin portion of OTA leading to 4-(R/S)-hydroxy-OTA -and 10-OH-OTA has been described from in vitro and in vivo studies as a minor metabolic route, and CYP2D6 together with CYP2B6 have been shown to be involved in the formation of these hydroxy metabolites [15,21,22,23,24]. In addition, 4-OH-OTA has been shown to be less toxic compared to OTA [25,26], and different kinetic properties are also expected for 4-OH-OTA inkling different affinities transporters involved in its renal excretion/re-uptake or enterohepatic circulation.

Since CYP2D6 is highly polymorphic, the existence of allelic variants with either an increased or reduced metabolic capacity to form hydroxy-OTA derivatives cannot be excluded a priori. In this case, an altered production of 4-OH-OTA compared to other OTA metabolites might have consequences on OTA’s TK and TD, and this would require further investigation. Broadly speaking, TK consequences associated with these specific CYP2D6 alleles have been extensively reported from the literature for a wide range of probe substrates, and consequences on TD have also been demonstrated, particularly for pharmaceuticals [6,9,27,28,29].

On this basis, the present work aims to provide a straightforward mechanistic approach to: I) semi-quantitatively estimate the metabolic activity of CYP2D6 variants to transform major CYP2D6 probe substrates (i.e., bufuralol and dextromethorphan) and OTA; II) assess the metabolic activity of poorly characterised CYP2D6 variants with regard to OTA hydroxylation. Shedding light on these inter-individual differences in the TK of OTA would provide a means to identify allelic variants worthy of further analysis which might cause a diverse susceptibility to OTA in certain human subpopulations. Finally, integrating such inter-individual differences will support the move towards mechanistically driven hazard assessment [30].

## 2. Results and Discussion

### 2.1. Assessing Model Efficacy 

This manuscript presents a 3D molecular modelling approach for the semi-quantification of the metabolic activity of CYP2D6 variants to metabolise well-described CYP2D6 probe substrates, namely dextromethorphan and bufuralol, as well as the mycotoxin OTA. In this context, the activities of CYP2D6 variants were shown to have low or null activity (CYP2D6*14A and CYP2D6*51) towards these probe substrates compared to that in the wild-type CYP2D6 (CYP2D6*1), which hydroxylates and demethylates bufuralol and dextromethorphan, respectively [27]. In contrast, CYP2D6*14A has no metabolic activity towards either probe substrates, while CYP2D6*51 had very low metabolic activity towards bufuralol and no activity reported for dextromethorphan [27]. OTA has been previously described as a minor substrate of CYP2D6 [22], but no data have been produced yet to investigate this hypothesis, particularly for different CYP2D6 variants.

**Figure 2:**
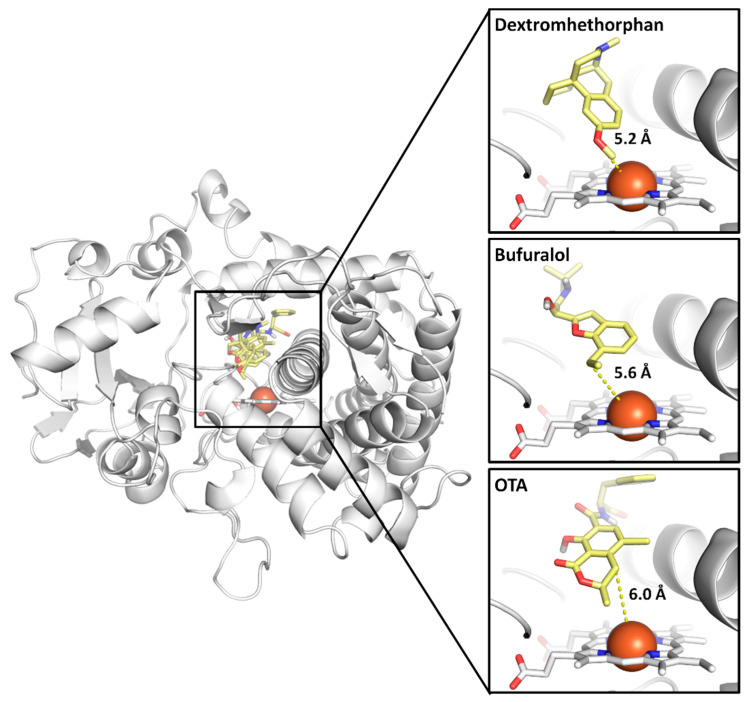
Docking poses of dextromethorphan, bufuralol and OTA within CYP2D6*1. Protein is represented in cartoon, while ligands and heme group are represented in sticks. Fe ion is represented in sphere. Yellow dashed lines indicate the distance between Fe and the atom undergoing the reaction.

In agreement with previous evidence describing the arrangement of atoms undergoing the reaction close to the Fe-heme (e.g., references [31,32]), the best-scoring docking pose for each ligand (dextromethorphan, bufuralol and OTA with 42.7, 51.7 and 59.34 GOLDScore units scored, respectively) was oriented towards the heme group (Figure 2). These results highlight the reliability of the current 3D in silico framework to provide plausible binding architectures. Interestingly, the atom, undergoing of the two probe substrates dextromethorphan and bufuralol, showed lower inter-atomic distances to the Fe-heme compared to OTA (Figure 2). These results are consistent with previous evidence describing OTA as a minor and non optimal substrate of CYP2D6 [22]. This evidence suggests that the inter-atomic distance between the atom undergoing the metabolic reaction and the Fe-heme provides a potential discriminant to distinguish CYP2D6 major probe substrates from CYP2D6 minor substrates that are poorly or not metabolised. In other words, the sub-optimal arrangement of OTA within the CYP2D6 isoform’s substrate-binding site, with a larger distance between the atom undergoing reaction and the Fe-heme compared to CYP2D6 major probe substrates, may provide a mechanistic explanation for the relatively low activity of CYP2D6 in transforming OTA to 4-OH-OTA.

**Figure 3:**
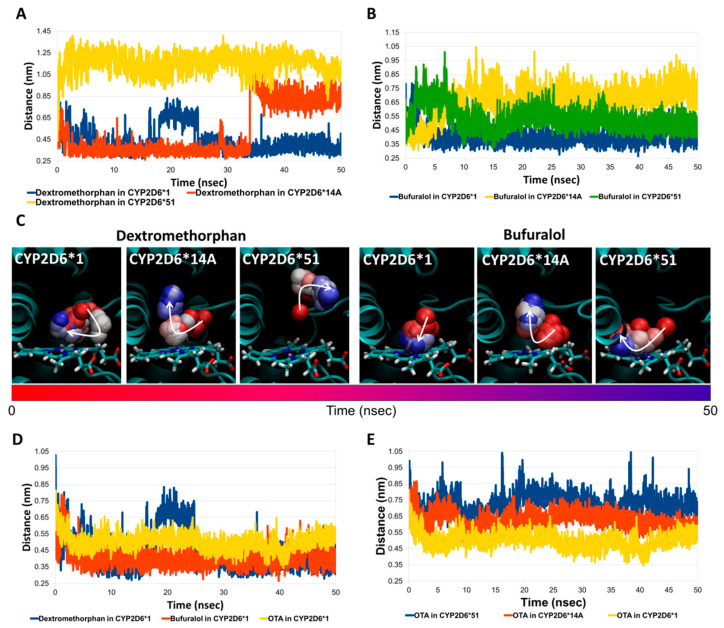
Molecular dynamics results on CYP2D6*1, CYP2D6*14A and CYP2D6*51. (**A**) Interatomic distances between dextromethorphan’s atom undergoing reaction and Fe-heme within CYP2D6*1, CYP2D6*14A or CYP2D6*51. (**B**) Interatomic distances between bufuralol’s atom undergoing reaction and Fe-heme within CYP2D6*1, CYP2D6*14A or CYP2D6*51. (**C**) Time-step representations of the trajectories of atom undergoing reaction (shown in sphere) of dextromethorphan or bufuralol within CYP2D6*1, CYP2D6*14A or CYP2D6*51. Proteins are represented in cartoon, while heme is represented in sticks. The from-red-to-blue colour switch indicates the stepwise changes of coordinates along the simulation. The white arrows retrace the direction of trajectories. (**D**) Interatomic distances between Fe-heme and the atom undergoing reaction of dextromethorphan, bufuralol or OTA within CYP2D6*1. (**E**) Inter-atomic distances between OTA’s atom undergoing reaction and Fe-heme within CYP2D6*1, CYP2D6*14A or CYP2D6*51.

In addition, the geometrical stability of dextromethorphan and bufuralol complexes with CYP2D6*1 was analysed over time (50 nsec) via molecular dynamic simulations and compared to those observed with CYP2D6*14A and CYP2D6*51 as respective variants with low or no activity against these two probe substrates. The mutated CYP2D6 variants in complex with dextromethorphan or bufuralol were derived from the respective complex with CYP2D6*1, as detailed in the Materials and Methods section. In this respect, the mutations of CYP2D6*14A and CYP2D6*51 were distant from the ligand-binding site (Figure S1; Supporting material) and were not considered relevant as mutations able to alter the binding architecture of the probe substrates, while general effects on the complex stability and dynamics were deemed more likely to happen. The root-mean-square deviation (RMSD) of protein C-alpha and the ligands’ atomic coordinates were analysed to measure the structural stability of complexes. The complexes between dextromethorphan or bufuralol with CYP2D6*1, CYP2D6*14A or CYP2D6*51 were found to be comparably stable within the considered timeframe and associated with similar trends in terms of protein’s C-alpha and ligand RMSD (Figure S2; Supporting material). However, distances between the atom undergoing the reaction for both probe substrates and the Fe-heme measured over time were significantly different in complex forms with CYP2D6*1, CYP2D6*14A and CYP2D6*51 (Figure 3A,B). More specifically, the atom undergoing metabolism within CYP2D6*1 was found to be closer to the Fe-heme compared to the respective complex with CYP2D6*14A or CYP2D6*51 for both compounds. Such a larger distance could provide a mechanistic basis for the inefficient transformation of these probe substrates by those CYP2D6 variants. For bufuralol, the atom undergoing the reaction was found to be consistently close to the Fe-heme during the whole simulation for CYP2D6*1, but not for CYP2D6*14A nor CYP2D6*51, where an early detachment from the Fe-heme was observed (Figure 3B,C). With regard to dextromethorphan in CYP2D6*1, the atom undergoing reaction was slightly further away for around 10 nanoseconds (from 15 to 25 nanoseconds) but got closer to the Fe-heme from 25 nanoseconds up to the end of simulation. Conversely, in CYP2D6*14A or CYP2D6*51, the CYP2D6-metabolised atom laid further away during the whole simulation (Figure 3A,C). These were associated with observed mean distances between the CYP2D6 Fe-heme and the dextromethorphan’s atom undergoing the reaction of 0.439 ± 0.002 nm with CYP2D6*1, and such a distance was significantly lower (*p* < 0.001) compared to those with CYP2D6*14A (0.518 ± 0.003) or CYP2D6*51 (1.157 ± 0.001 nm), respectively. Here, it is worth noticing that for CYP2D6*14A, the distance between the dextromethorphan’s atom undergoing the reaction and Fe-heme increased from 34 nanoseconds, while in complex with CYP2D6*51, such an increase was observed at the start of simulation. These results could suggest that the mutations of CYP2D6*14A may exert a delayed effect on the binding architecture of dextromethorphan, whereas the mutations of CYP2D6*51 exert a more rapid effect. With regard to bufuralol, the distance to the Fe-heme observed in complex with CYP2D6*1 was 0.407 ± 0.001 nm, which was significantly lower (*p* < 0.001) compared to that observed in complex with CYP2D6*14A or CYP2D6*51 (0.659 ± 0.002 or 0.535 ± 0.001 nm, respectively). Of note, the very low activity of CYP2D6*51 against bufuralol was previously described [27], and our results show that the distance to the Fe-heme in CYP2D6*51 was larger compared to that observed in complex to CYP2D6*1 but significantly smaller (*p* < 0.001) than that observed in complex to CYP2D5*14A, which showed no activity against bufuralol [27]. In this line of interpretation, keeping in mind the low expected capability of CYP2D6 to transform OTA [22], the distance between the atom undergoing a reaction of OTA to Fe-heme in CYP2D6*1 was measured and compared to those observed for dextromethorphan and bufuralol. Interestingly, the complex with dextromethorphan or bufuralol showed similar distance values, while OTA recorded distance values slightly higher over time (Figure 3D) with a significantly higher average distance (*p* < 0.001; 0.501± 0.001 nm) compared to the two probe substrates (i.e., 0.439 ± 0.002 and 0.407 ± 0.001 nm for dextromethorphan and bufuralol, respectively).

Overall, the results presented here highlight the reliability of the above-described workflow, particularly in the investigation of the distance between an atom undergoing metabolism and the CYP2D6 Fe-heme. In addition, they also provide a straightforward and effective structural discriminant to semi-quantitatively estimate the likelihood of ligands to act as major probe substrates or minor substrates of CYP2D6 and its variants.

The complex between OTA and CYP2D6*14A or CYP2D6*51 was also investigated, but in neither of the two complexes was OTA found to be arranged in a favourable position for metabolism (i.e., with a lower inter-atomic distance between the atom undergoing reaction and Fe-heme) compared to the complex with the wild-type CYP2D6*1 (Figure 3E). Hence, CYP2D6*14A and CYP2D6*51 were associated with poor and possibly even reduced metabolic activity towards OTA compared to that for CYP2D6*1. This conclusion is based on the larger distance between the atom undergoing the reaction and the CYP2D6 Fe-heme (i.e., 0.628 ± 0.001 and 0.720 ± 0.001 in CYP2D6*14A and CYP2D6*51, respectively, versus 0.501± 0.001 nm in CYP2D6*1).

### 2.2. Analysis of Uncharacterised CYP2D6 Variants

**Figure 4:**
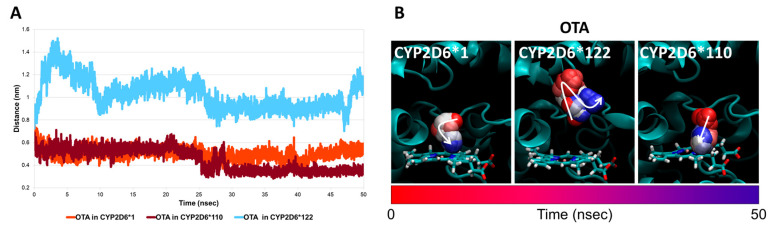
Molecular dynamics results of CYP2D6*1, CYP2D6*110 and CYP2D6*122. (**A**) Interatomic distances between OTA’s atom undergoing reaction and Fe-heme within CYP2D6*1, CYP2D6*110 or CYP2D6*122. (**B**) Time-step representations of the trajectories of atom undergoing reaction (shown in sphere) of OTA within CYP2D6*1, CYP2D6*110 or CYP2D6*122. Proteins are represented in cartoon, while heme is represented in sticks. The from-red-to-blue colour switch indicates the stepwise changes of coordinates along the simulation. The white arrows retrace the direction of trajectories.

Based on the above considerations, the measurement of inter-atomic distances between the atom undergoing reaction and the Fe-heme has been described as a discriminant feature to estimate the likelihood of ligands to act as major or minor substrates for CYP2D6*1 and its variants. Therefore, two poorly characterised CYP2D6 variants, i.e., CYP2D6*110 and CYP2D6*122, were also investigated for their capacity to allow a proper arrangement of OTA and its atom with the Fe-heme to form 4-OH-OTA. As shown in Figure 4, two opposite trends were described for OTA within these two CYP2D6 variants. In particular, the interaction with CYP2D6*122 did not suggest a proper arrangement of the atom undergoing the reaction, considering its marked and early detachment from the Fe-heme. This movement was also found to be associated with the overall instability of the OTA geometry within the substrate-binding pocket, as demonstrated by the RMSD analysis (Figure S3, Supporting material). It is important to note that the results collected suggested a weaker interaction between OTA and the CYP2D6*122 variant compared to the wild-type CYP2D6*1. This result reflects earlier detachment of the atom undergoing metabolism from the Fe-heme and the overall geometric instability of the OTA binding architecture. On this basis, CYP2D6*122 was not considered to efficiently metabolise OTA to 4-OH-OTA with an expected reaction yield lower than that for CYP2D6*1. 

Conversely, OTA in complex with CYP2D6*110 showed a more stable geometry within the substrate pocket (Figure S3; Supporting material). In addition, the atom undergoing the reaction was also found close to the Fe-heme along the whole simulation, and the interaction distance was closer compared to that observed for the CYP2D6*1 complex from 25 nanoseconds up to the end of the simulation. Indeed, the mean inter-atomic distance recorded for the OTA-CYP2D6*110 complex was significantly lower (*p* < 0.001) compared to that observed for the OTA-CYP2D6*1 (0.448 ± 0.001 and 0.501 ± 0.001, respectively).

The primary sequence of CYP2D6*1 differed from CYP2D6*110 by only one amino acid substitution (G445R) close to the heme-binding site, and a close analysis of the protein revealed that the G445R mutation was not likely to cause interferences in heme interaction. Conversely, such a mutation appeared to stabilise the heme-protein interaction, as shown by RMSD analysis. Hence, more stable interaction of the heme for CYP2D6*110 can be shown compared to that with CYP2D6*1 (Figure S4A; Supporting material). This effect was likely due to additional cation–π contributions, which are well-documented stabilising interactions in biological systems (e.g., ref. [33]). These interactions occur between the guanidinium group of the arginine’s side chain and the proximal ethenyl and pyrrolic group of the heme (Figure S4B). The hydrophobic neck of Arg could also contribute with favourable hydrophobic/hydrophobic interactions in line with previous data describing the capacity of polar amino acids with hydrophobic necks to bring such stabilizing interactions (e.g., ref. [34]). With regard to the actual expression of the CYP2D6*110 variant, data are still scarce and therefore, in agreement with our previous study [30], the effect of the CYP2D6*110 mutation on protein stability was assessed using the PremPS method [35]. This method accurately tests the effects of missense mutations on protein stability and estimates the unfolding Gibbs free energy changes (ΔΔG) [35]. The G445R missense mutation recorded a predicted unfolding free energy change (ΔΔG) of 0.9 kcal mol^−1^. Previous studies demonstrated that ΔΔG < 1 kcal/mol are associated with a theoretical limited effect on protein stability [35]. Therefore, the mutation of CYP2D6*110 was estimated to be compliant with its actual expression. 

Taken together, these results suggested a possible increased metabolic activity for CYP2D6*110 to transform OTA to 4-OH-OTA compared to that for CYP2D6*1. This outcome was in contrast with the activity of CYP2D6*110 previously calculated through bioinformatics and for which impaired substrate–enzyme interaction was predicted [36]. Such a prediction was based on a multiple-sequence alignment (MSA) analysis between CYP2D6*1 and some homologous sequences describing a full conservation of G at the position 445. This result provides a piece of evidence that such substitutions are unlikely in functional CYPs. However, the analysis was based on sequence databases that are out of date (e.g., UniProtKB/UniRef100 Release 2011_12 updated to 14 December 2011) [36]. Hence, a further MSA (Supporting material) was performed using the most up-to-date set of sequences available in the UniProt database (https://www.uniprot.org; last database accessed on 25 November 2021) [37]. This MSA included 1635 sequences homologous to CYP2D6*1 and showed that G445 was conserved in 95% of the sequences considered, with 5% of the sequences (80 sequences) showing substitutions with bulkier and more polar amino acids including Ala, Ser, Tyr, Phe, Asp and Leu (Table S1 and Figure S5; Supporting material). These 5% of homologous sequences include functional CYPs such as the human CYP39A1 and the chicken CYP1A2. Moreover, two of the active homologs had crystallographic structures available in Protein Data Bank (UniProt ID P23295 with PDB code 1CL6 and Q9K498 with PDB code 3DBG) showing G445A and G445S substitutions, respectively. These structures allowed a close visual inspection of the heme binding architecture which revealed that, in both cases, the substitution was likely to contribute to protein–heme binding via hydrophobic/hydrophobic interaction (Figure S4; Supporting material) in a similar fashion to the G445R substitution in CYP2D6*110. A similar contribution was also observed for the CYP2D6 homolog with a PDB structure 5LI6 (UniProt ID P9WPN9). For CYP2D6*1, V374 surrounds the heme group, while for the 5LI6 structure, a substitution of Gly with Lys was observed. In these structures, the organisation and interactions with the heme group were very similar to that observed for the G445R mutation in the CYP2D6*110 structure (Figure S4; Supporting material). Moreover, Li and co-workers [38] explained that the heme-binding motif (i.e., GX[HR]XC[PLAV]G) is generally well conserved in heme-binding proteins, although a certain degree of variability for the position +2 has been observed in crystallographic studies, with Ala, Ser or Lys substituting Gly (i.e., the two positions downstream the Cys coordinating Fe-heme and corresponding to G445). In addition, selenomethionine (MSE) was also present at the same position of G445 (e.g., in the PDB structure 3CQV) and formed the same architecture and interactions described for G445R (Figure S4E, Supporting material).

Taken together, these results suggested that the position G445 (corresponding to the position +2 in the heme-binding motif) can be substituted with other residues while keeping the CYP2D6 variants functional and the heme–protein complexes stable, although it has a relatively high degree of conservation. Therefore, the substitution G445R may retain a certain degree of activity in the CYP2D6*110 variant, and this may result in higher reaction rates towards OTA compared to that for CYP2D6*1. Further analyses assessing the impact of CYP2D6 variants on OTA biotransformation are warranted, particularly with regard to their potential impact on the potential TK and TD. For example, CYP2D6*110 may produce larger amounts of 4-OTA-OTA and may be associated with a more effective clearance of OTA. However, protein expression, stability, turnover, heme’s incorporation and potential activity in cells and living organisms should be assessed in future studies to ultimately characterise the effects of the G455R substitution. 

## 3. Conclusions

In line with current proposals to support the use of New Approach Methodologies (NAMs) in human risk assessment of chemicals [39], the pipeline described here provides a means for the systematic analysis of the impact of CYP polymorphisms on food xenobiotics. In particular, the impact of CYP2D6 allelic variants polymorphisms on the biotransformation of OTA has been showcased as a proof-of-principle study. Notably, in the past, CYP2D6 was considered to have a major role in OTA toxicity based on the apparent increased risk of extensive CYP2D6 metabolisers to develop OTA-related diseases (e.g., urinary tract tumours and Balkan endemic nephropathy (BEN)) [40,41]. This hypothesis was then refuted, and CYP2C has been described as an important OTA biotransforming enzyme with possible consequences on its toxicity [42,43,44]. In addition, CYP polymorphisms were associated with an increased risk of BEN due to allele-specific OTA biotransformation [45,46]. Overall, this line of evidence points to the possible relevance of CYP isoforms to OTA toxicity and, although CYP2D6 has a minor role in OTA biotransformation and toxicity at a population level, the TK and TD consequences of the SNPs described in this work should be further evaluated in hazard assessment to enable the integration of data from low-frequency SNPs and inter-individual differences in CYP2D6-mediated biotransformation [39]. This pipeline is also relevant to screen food chemicals as potential CYP2D6 substrates. Although this information is generally known for pharmaceuticals, it is often lacking for chemicals that are present in the food chain, including food additives, pesticide active substances, natural toxins and contaminants. It is foreseen that such knowledge will provide a means to take into account human variability in kinetics for the CYP2D6 isoform, particularly to relate the consequences of metabolism and the potential sensitivity of the different human CYP2D6 phenotypes to chemical toxicity. For example, PMs may be potentially sensitive to chemicals for which CYP2D6 is involved in detoxification reactions, whereas EMs and UMs may be potentially sensitive to chemicals for which CYP2D6 is involved in bioactivation reactions.

It is worth noting that previous molecular modelling studies have addressed CYP2D6 polymorphisms while providing valuable workflows to deepen system biophysics and biomechanics [10,11,12,47]. However, the inherent complexity of those analyses and outcomes renders their routine application unfeasible for a systematic analysis of wide sets of CYP variants and chemicals. Conversely, we demonstrated that using a straightforward mechanistic parameter, such as the inter-atomic distance between the atom undergoing the reaction and the Fe-heme, allows one to discriminate ligands in a semi-quantitative fashion for their likelihood to undergo biotransformation by CYP2D6 variants. This would warrant a fit-for-purpose pipeline which can be extended to a tool allowing for the bulk analysis of many compounds to ultimately integrate CYP2D6 inter-phenotypic differences in chemical risk assessment. 

## 4. Materials and Methods

**Figure 5:**
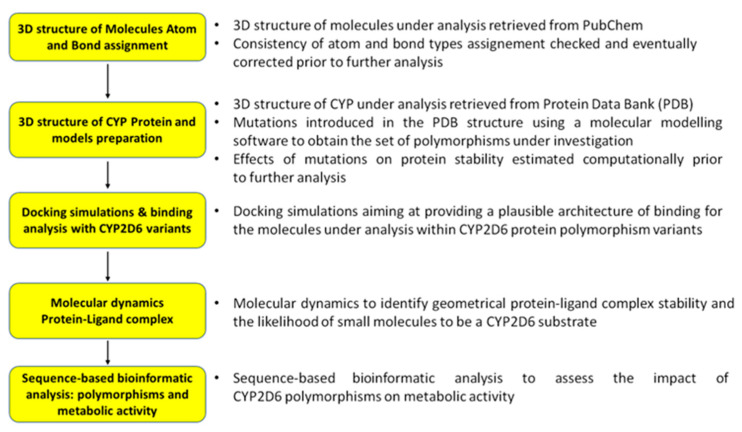
Flowchart of key methodological steps for the ligand-CYP2D6 analysis.

A flowchart highlighting the key methodological steps for the ligand-CYP2D6 analysis is illustrated in Figure 5. 

### 4.1. Data Source 

Dextromethorphan (CAS code 125-71-3) and (-)-bufuralol (CAS code 64100-62-5), as CYP2D6 probe substrates, were analysed together with OTA (CAS code 303-47-9) as low-molecular-weight molecules. All 3D structures were retrieved from the PubChem database (https://pubchem.ncbi.nlm.nih.gov, accessed on 13 July 2021) [48] in the 3D structure data file format (.sdf) format with the following respective PubChem CIDs: 442530 (dextromethorphan), 5360696 (bufuralol) and 11448378 (OTA). The consistency of atom and bond type has been checked with UCSF Chimera software (version 1.15) [49] prior to further analysis. The 3D model of the wild-type human CYP2D6*1 (Uniprot code P10635) was derived from the crystallographic structure retrieved in the .pdb format from the Protein Data Bank (PDB; https://www.rcsb.org, accessed on 13 July 2021) [50] with the PDB code 4WNW [32]. Since the crystallographic structures for the CYP2D6 variants included here (i.e., CYP2D6*14A, CYP2D6*51, CYP2D6*110 and CYP2D6*122) were not available, an in silico approach was applied to generate CYP2D6 variants with single-amino-acid mutations (see below).

### 4.2. Model and Ligands Preparation 

The consistency of all atom and bond type assignments, geometries of ligands and CYP2D6 structures were visually checked with UCSF Chimera software (version 1.15) [49]. The carboxylic group of OTA was set as deprotonated, while amino and carboxy terminals of CYP2D6 were set as deprotonated and protonated, respectively. The CYP2D6 model was derived from chain A with an unresolved non-terminal region (residues 142–146). This missing region was then modelled using the “model loop/refine structure” of Modeller (version 10) [51] interfaced with UCSF Chimera software (version 1.15) [49] prior to further analysis. Single-amino-acid mutations to generate CYP2D6 variants were introduced while replacing specific amino acid side-chains using the Structure Editing/Rotamer tool of UCSF Chimera software (version 1.15) [30]. Then, from multiple computed rotamers, the rotamer with the highest computed probability of occurrence was selected in agreement with our previous study [30]. The CYP2D6 variants included here were CYP2D6*14A (P34S, G169R, R296C and S486T), CYP2D6*51 (R296C, E334A and S486T), CYP2D6*110 (G445R) and CYP2D6*122 (V370I). In addition, the stability of the CYP2D6*110 variant was calculated using the PremPS method [35], consistently with a previous study [30]. This method provides a specific and accurate assessment of the impact of missense mutations on protein stability through the implementation of a random forest regression scoring function to estimate unfolding Gibbs free energy changes (ΔΔG). 

### 4.3. Docking Analysis 

The docking analysis aimed to provide a plausible binding architecture for the chemicals under investigation. This was performed using the GOLD software (version 2021), which has already shown high reliability for the computation of protein–ligand interactions [52,53]. The binding site was defined within a 10-angstrom radius sphere around the centroid of the substrate-binding site. The docking protocol was set according to previous studies for which ligands were kept fully flexible and protein semi-flexible, allowing polar hydrogens to rotate freely [54]. As a minor modification, the internal scoring function GOLDScore was used, as it was optimised for the prediction of ligand-binding positions according to the manufacturer declaration (https://www.ccdc.cam.ac.uk, accessed on 13 July 2021). Finally, for each ligand, the “region constraint” option was selected together with an option avoiding the generation of docking poses when the constraint was physically impossible. 

### 4.4. Molecular Dynamics 

Molecular dynamics aimed to investigate the effects of CYP2D6 missense mutations on the geometry of protein–ligand complexes over time. These were performed using GROMACS (version 5.1.4) [55], while ligands were parametrised with a CHARMM27 all-atom force field [56]. The hydrogen database was modified according to previous works [57,58] to parameterise the heme group. Input structures were solvated with SPCE waters in a cubic periodic boundary condition, and counter ions (Na^+^ and Cl^−^) were added to neutralise the system. Prior to running simulations, each system was energetically minimised to avoid steric clashes and to correct improper geometries using the steepest descent algorithm with a maximum of 5000 steps. Subsequently, each system underwent isothermal (300 K, coupling time 2 psec) and isobaric (1 bar, coupling time 2 psec) 100 psec simulations before running 50 nsec simulations (300 K with a coupling time of 0.1 psec and 1 bar with a coupling time of 2.0 psec). 

### 4.5. Statistical Analysis 

The average distance observed between the atom undergoing reaction of each ligand and the Fe-heme in each complex in the molecular dynamic simulations was calculated with SPSS IBM (v. 23.0, SPSS Inc., Chicago, IL, USA). Overall, 5000 frames were analysed for each complex. Independent Student’s *t* tests (α = 0.05) were used to compare the pair-wise average distance of each ligand within CYP2D6*1 and those generated with the other variants considered in this study. Values are expressed as mean ± standard error (SE). 

### 4.6. Multiple-Sequence Alignment Analysis 

The subset of reviewed sequences belonging to the CYP450 family (pfam identifier PF00067) stored in the UniProt database (https://www.uniprot.org, with last database accessed on 25 November 2021) [37] were downloaded in the FASTA format (1635 sequences in total). Then, a multiple-sequence alignment (MSA) was run using Clustal O version 1.2.4 (https://www.ebi.ac.uk/Tools/msa/clustalo, accessed on 25 November 2021) [59] with default parameters. The output was downloaded and stored locally for further analysis.

## Supplementary Materials

The following supplementary materials are available online at https://www.mdpi.com/article/10.3390/toxins14030207/s1, Figure S1: Distribution of mutations on CYP2D6*14A and CYP2D6*51 compared to CYP2D6*1; Figure S2: Results of molecular dynamic simulation of dextromethorphan and bufuralol within CYP2D6*1, CYP2D6*14A and CYP2D6*51; Figure S3: RMSD analysis of OTA in complex with CYP2D6*1, CYP2D6*122 or CYP2D6*110; Figure S4: Analysis of CYP2D6*110 in comparison to other CYPs; Figure S5: Multiple-sequence alignment (MSA) logo; Table S1: List of UniProt sequences from MSA with substitutions at the position 445 of CYP2D6.
