# Supplementary material for: A Computational Understanding of Inter-Individual Variability in CYP2D6 Activity to Investigate the Impact of Missense Mutations on Ochratoxin A Metabolism
Source: Toxins (Basel). 2022 Mar 14;14(3):207. doi: 10.3390/toxins14030207 (PMC8950366; doi:10.3390/toxins14030207)
Supplement: Supplementary file 1 [file toxins-14-00207-s001.zip › toxins-1599668-supplementary.pdf]

Supplementary Materials: A Computational Understanding of Inter-Individual Variability in CYP2D6 Activity to Investigate the Impact of Missense Mutations on Ochratoxin A Metabolism

Jean Lou C. M. Dorne, Martina Cirlini, Jochem Louisse, Lorenzo Pedroni, Gianni Galaverna and Luca Dellafiora

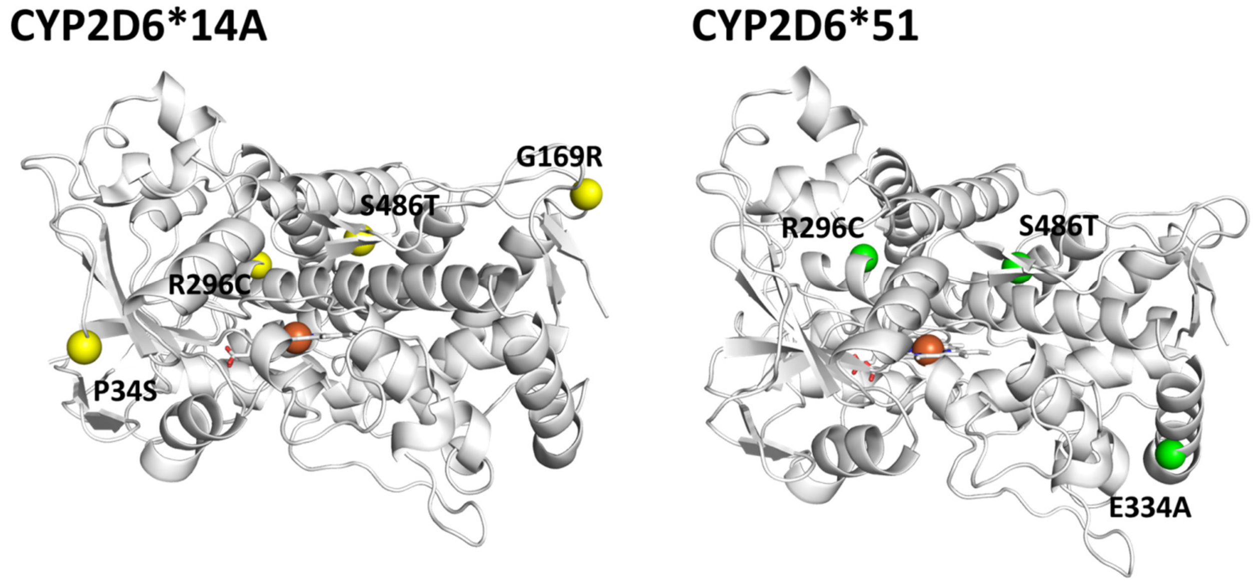

Figure S1. Distribution of mutations on CYP2D6*14A and CYP2D6*51 compared to CYP2D6*1, as per PharmVar Database (https://www.pharmvar.org, accessed on 4 October 2021). The red sphere indicates the Fe-heme and identifies the position of substrate binding site. Mutated positions are represented in yellow and green spheres on CYP2D6*14A or CYP2D6*51 structure, respectively.

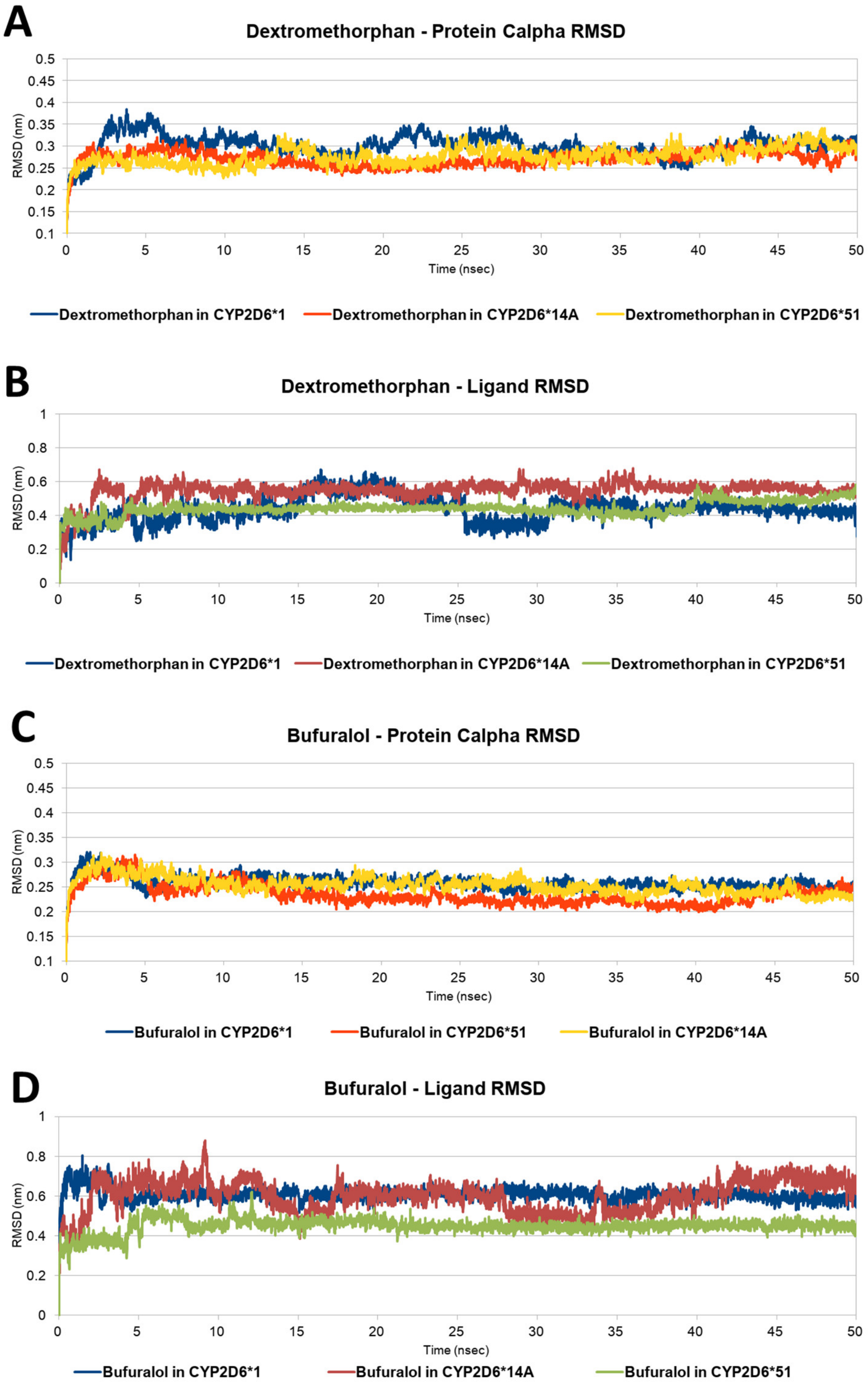

Figure S2. Results of molecular dynamic simulation of dextromethorphan and bufuralol within CYP2D6*1, CYP2D6*14A and CYP2D6*51. (**A**) Protein C-alpha RSMD of dextromethorphan within CYP2D6*1, CYP2D6*14A or CYP2D6*51. (**B**) Ligand RSMD of dextromethorphan within CYP2D6*1, CYP2D6*14A or CYP2D6*51. (**C**) Protein C-alpha RSMD of bufuralol within CYP2D6*1, CYP2D6*14A or CYP2D6*51. (**D**) Ligand RSMD of bufuralol within CYP2D6*1, CYP2D6*14A or CYP2D6*51.

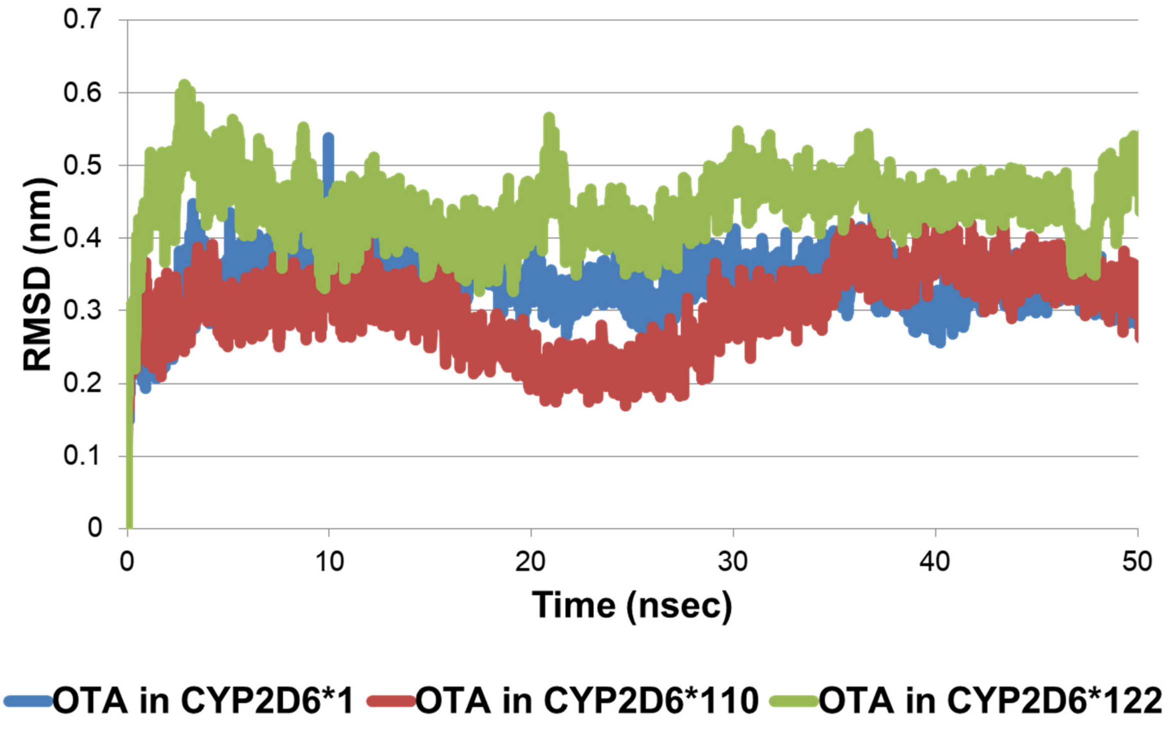

Figure S3. RMSD analysis of OTA in complex with CYP2D6*1, CYP2D6*122 or CYP2D6*110.

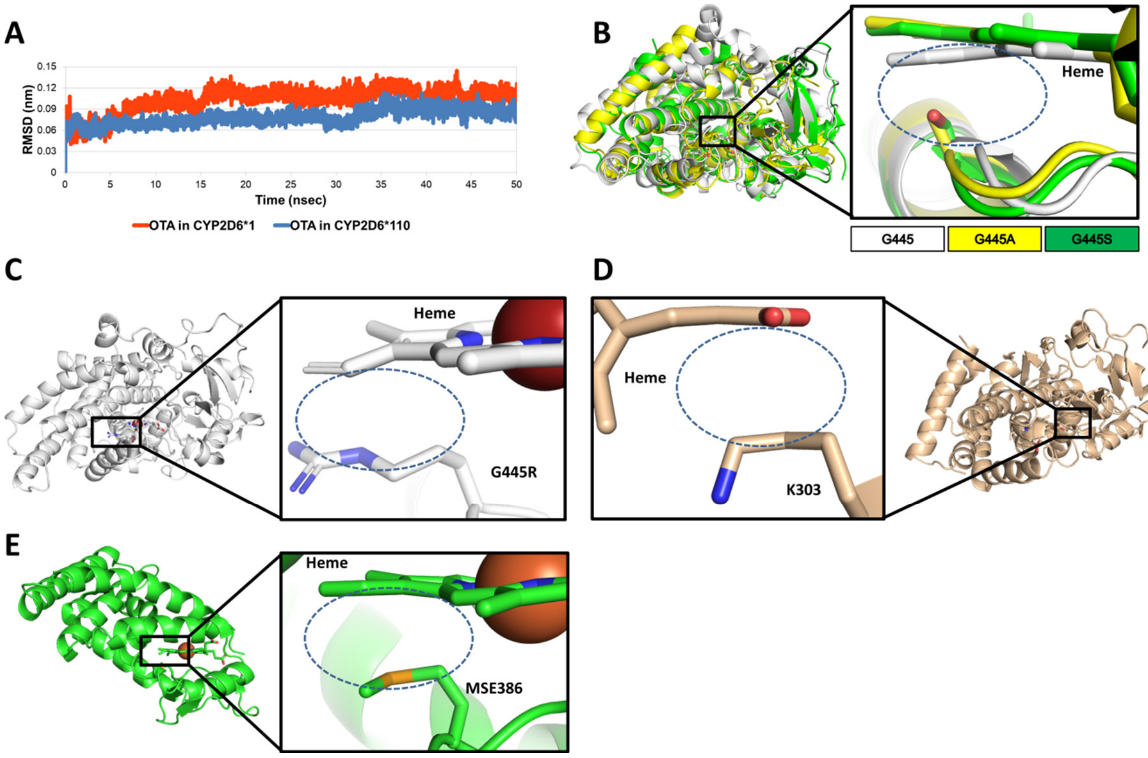

Figure S4. Analysis of CYP2D6*110 in comparison to other CYPs. Proteins are represented in cartoon while heme group and amino acid side chains are represented in sticks. Dashed rings indicate the region likely interested by cation–π or hydrophobic/hydrophobic contributions to the heme binding. Fe ion is represented by sphere. (**A**) RMSD plot of heme group in OTA-CYP2D6*1 or OTA-CYP2D6*110 complex. (**B**) CYP2D6*1 (shown in white; PDB code 4WNW chain A) overlapped to two homologs with Ala (shown in yellow) or Ser (shown in green) substitution at the position 445 (PDB code 1CL6 chain A and 3DBG chain A, respectively). (**C**) Detail of G445R mutation in CYP2D6*110. (**D**) Detail of Lys-heme interaction in an CYP homolog to CYP2D6*1 (PDB code 5LI6). (**E**) Detail of MSE (selenomethionine)-heme interaction in the structure having PDB code 3CQV.

Table S1. List of UniProt sequences from MSA with substitutions at the position 445 of CYP2D6.

Uniprot Entry ID	Substitution	Uniprot Entry ID	Substitution
Q01741	445G > A	I3PLR0	445G > A
A0A1D6HSP4	445G > A	Q9CA60	445G > A
A0A1D6F9Y9	445G > A	Q9CA61	445G > A
O65787	445G > A	Q5KTN3	445G > L
Q9LVD2	445G > A	A0A0C6DUU3	445G > A
Q9LIP3	445G > A	Q0UK49	445G > A
Q9LIP4	445G > A	C9K202	445G > A
Q9LIP6	445G > A	Q9V979	445G > A
Q9LIP5	445G > A	Q9VCW1	445G > A
O65782	445G > A	Q9W223	445G > A
A0A068Q721	445G > A	Q964Q7	445G > A
Q9STK8	445G > A	Q27698	445G > A
Q9STK7	445G > A	Q54E98	445G > S
Q9STK9	445G > A	D1MX85	445G > A
Q9STL1	445G > A	O00061	445G > A
Q9STL2	445G > A	A0A411KZY9	445G > A
A0A084R1J2	445G > S	Q9V4T3	445G > A
A0A068Q7V0	445G > A	B6HJU5	445G > A
Q501D8	445G > A	K4CF70	445G > A
O81345	445G > A	B3LF83	445G > A
O81346	445G > A	Q9ZSY9	445G > A
Q43135	445G > A	A0A0N9HKQ7	445G > A
Q9M7B7	445G > A	B8N8R3	445G > A
Q9M7B8	445G > A	A2QK67	445G > A
Q5E980	445G > E	A0A1Y1C7S2	445G > Y
Q6UW02	445G > E	B8NM64	445G > A
Q8BKE6	445G > E	Q9K498	445G > S
Q6P7D4	445G > E	A0A0C1E1L9	445G > A
Q9T093	445G > A	B8NJH2	445G > A
Q9NGX9	445G > A	Q5ATG9	445G > A
Q12599	445G > A	P0DO32	445G > A
Q00616	445G > A	A0A482NAF7	445G > A
P23295	445G > A	A0JJT8	445G > A
Q9ZGH8	445G > V	A0A167LUS5	445G > A
Q9L4U5	445G > F	A0A1V1FNZ5	445G > A
P9WPL6	445G > A	Q9VS78	445G > A
P9WPL7	445G > A	B8NR71	445G > A
P58047	445G > D	Q1ZXG3	445G > F
Q9FH67	445G > A	Q54D38	445G > A
A0A0N7F297	445G > A	Q9NYL5	445G > A

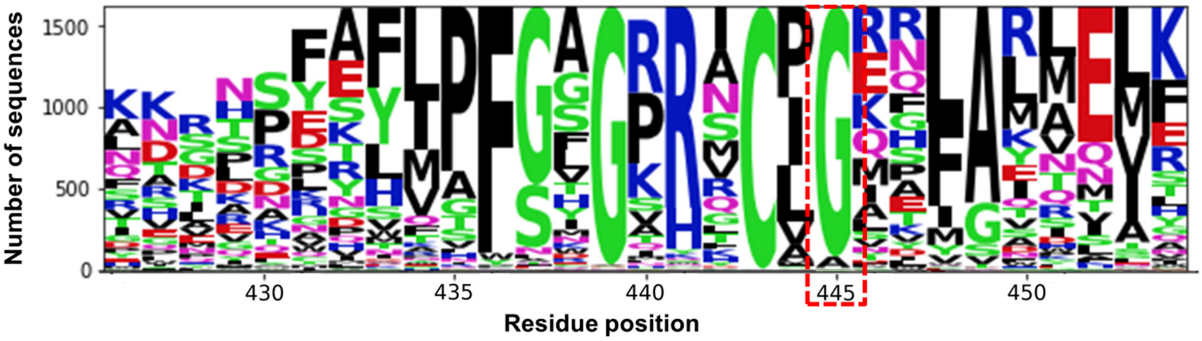

Figure S5. Multiple sequence alignment (MSA) logo. Only the region close to the 445 position is shown (residues 425–455). The character size is proportional to the amino acid occurrence in the MSA. The position 445 is highlighted with a red box.
